# Targeting stearoyl-CoA desaturase 1 to repress endometrial cancer progression

**DOI:** 10.18632/oncotarget.24304

**Published:** 2018-01-24

**Authors:** Weihua Li, Huimin Bai, Shiping Liu, Dongyan Cao, Hongying Wu, Keng Shen, Yanhong Tai, Jiaxin Yang

**Affiliations:** ^1^ Department of Obstetrics and Gynecology, Peking Union Medical College Hospital, Chinese Academy of Medical Sciences and Peking Union Medical College, Wangfujing, Beijing 100730, China; ^2^ Department of Obstetrics and Gynecology, Beijing Chao-Yang Hospital Affiliated to Capital Medical University, Beijing 100020, China; ^3^ Departments of Obstetrics and Gynecology, Peking University First Hospital, Beijing 100034, China; ^4^ Institute of Radiation Medicine, The Chinese Academy of Medical Sciences, Tianjin 300192, China; ^5^ Department of Pathology, The Affiliated Hospital of Military Medical Science Academy of Chinese People’s Liberation Army (307 Hospital of Chinese People’s Liberation Army), Beijing 100071, China

**Keywords:** stearoyl-CoA desaturase, endometrial cancer, therapeutic target, cell growth, cell death

## Abstract

Stearoyl-CoA desaturase 1 (SCD1) is an established molecular target in many primary tumors including breast, lung, pancreatic, colon and hepatocellular carcinomas. However, its potential role in supporting endometrial cancer growth and progression has not yet been determined. In this study, we evaluated the value of SCD1 as a candidate therapeutic target in human endometrial cancer. Compared with secretory and post-menopausal endometrium, SCD1 was highly expressed in normal endometrium of proliferative phase, endometrial hyperplasia and endometrial carcinoma, while was absent or low expression in non-malignant control stromal cells and adjacent normal endometrium. Knockdown of SCD1 significantly repressed endometrial cancer cell growth and induced cell apoptosis. Both short hairpin RNA targeted knockdown and chemical inhibitor of SCD1 suppressed the foci formation of AN3CA, a metastatic endometrial cell line. Xenograft model further demonstrated that reduced SCD1 expression impaired endometrial cancer growth *in vivo*. Taken together, these findings indicate that SCD1 is a potentially therapeutic target in human endometrial cancer. Inhibiting lipid metabolism in cancer cells would be a promising strategy for anti-cancer therapy.

## INTRODUCTION

Endometrial carcinoma (EC) is the most common gynecologic malignancy in Europe and North America, and its incidence and mortality is gradually on the rise worldwidely. In the United States, the estimated new cases and deaths from EC in 2017 are 61 380 and 10 920, respectively [1]. It is well-documented that obesity is a high risk factor for endometrial cancer. There is a linear relationship between body mass index and risk of endometrial cancer among postmenopausal women. However, the nature of this relationship remains to be fully elucidated [2]. Obese women have a 2∼4 times greater risk of developing EC compared to women of normal weight, regardless of menopausal status [3–5].

Currently, obesity is a problem of epidemic proportions in many developed nations, the incidence of endometrial cancer is expected to keep growing. It is reported that the onset of obesity and pathogenesis of endometrial cancer may share same metabolic and biological pathways [6]. Obesity and its metabolic complications are associated with upregulated expression/activity of stearoyl-CoA desaturase-1 (SCD1) [7], a fatty acid desaturase in the endoplasmic reticulum. SCD1 is a rate-limiting enzyme that converts saturated fatty acids (SFAs) to monounsaturated fatty acids (MUFAs) in cellular *de novo* fatty acid synthesis. Increased content of MUFAs were observed in a variety of transformed cells and cancers [8–9], suggesting that enhanced fatty acid desaturation is an important process in cancer cells.

Elevated expression and activity levels of SCD1 have been reported in various human cancers, such as hepatocellular carcinoma, renal clear cell carcinoma, lung cancer and breast cancer [10–14]. The development and maintenance of cancer demand a massive production of lipid biomolecules to support cell proliferation and survival signaling pathways.SCD1 deficiency and/or inhibition leads to a slower rate of cell proliferation, a loss of anchoring growth and higher rates of apoptosis in various cancer cells [8, 15]. An increasing number of experimental studies have demonstrated that SCD1 is a transcriptional target of sterol regulatory element-binding protein 1 (SREBP1) and Peroxisome Proliferator Activated Receptor (PPAR) [16–22].

Although many lipogenic enzymes have been shown to be upregulated in EC, the potential role and mechanism of SCD1 in this malignancy remains\largely unknown. Given the importance of SCD1 in governing the production of phospholipids, which are the “building blocks” of tumor cells, we studied the correlation between SCD1 and SREBP1, and explored the therapeutic value of targeting SCD1 in endometrial cancer. Our results showed that SCD1 was overexpressed in EC, and its expression level was positively correlated with the level of SREBP1, the transcription regulator of SCD1. Importantly, knockdown of endogenous SCD1 reduced the lipid production, hampered cellular proliferation, reduced colony formation, and increased cell apoptosis. Furthermore, *in vivo* animal experiments showed that downregulation of SCD1 impaired tumor growth. Taken together, these observations demonstrate that SCD1 plays a vital role in EC celluar proliferation, supporting our hypothesis that enhanced SCD1 as a result of SREBP1 over-activation may contribute to EC progression through supplying tumor cells with abundant MUFA.

## RESULTS

### SCD1 is upregulated in endometrial carcinoma compared to its adjacent normal tissue

**Figure 1:**
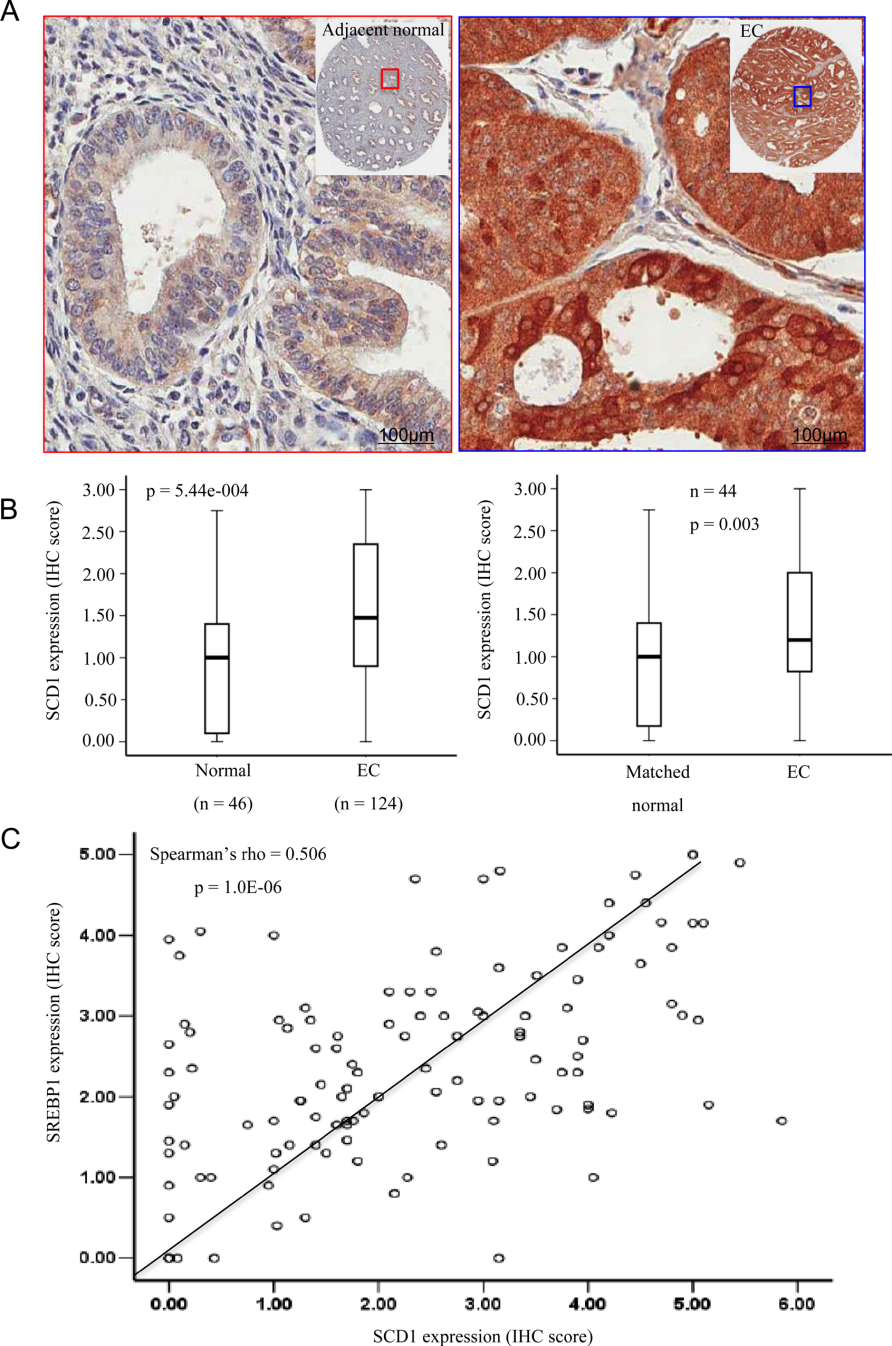
SCD1 is overexpressed in endometrial cancer (EC) determined by immunohistochemistry (IHC) (**A**) SCD1 was much highly expressed in endometrial cancer tissues than in matched adjacent normal endometrium. (**B**) Boxplot of IHC staining score for SCD1 expression in whole cell in all endometrial cancer specimens and matched non-cancerous tissues or normal endometrium. (**C**) Scatter diagram of IHC staining score for SCD1 and SREBP1 expression in endometrial cancer. The expression level of SCD1 is correlated with SREBP1 expression.

Several studies have shown that fatty acid synthase (FASN), a key lipogenic enzyme catalysing the terminal steps in the *de novo* biogenesis of fatty acids, and SREBP1 are overexpressed in endometrial cancer [23, 24]. However, the underlying mechanism is still unclear [25]. Activation of FASN, SCD1 and other lipogenic genes are directly regulated by SREBP1, a major transcription factors that regulate genes involved in lipid metabolism [26, 27]. By providing MUFA, SCD1 governs the production of phospholipids, the major component of cell membrane. Therefore, we investigated whether increased SCD1 expression contributed to EC progression as a result of increased SREBP1 expression and enhanced transactivation. To detect the expression level of SCD1, we performed immunohistochemical staining on formalin-fixed, paraffin-embedded sections using anti-SCD1 antibody. The cytoplasmic expression levels of SCD1 were scored. As shown in Figure 1, SCD1 was significantly upregulated in endometrial carcinoma compared to its adjacent normal tissue. As we had shown that SREBP1 was overexpressed in the same set of clinical EC specimens [28], we compared the expression of SREBP1 and SCD1, which showed significant correlation (rho = 0.506, *p* = 1.0E-06). These observations indicate that SCD1 overexpression could be the consequence of SREBP1 abnormal expression and activation, suggesting a role of SCD1 in mediating dysregulated function of SREBP1 in EC.

### SCD1 expression is increased in proliferative and hyperplasia endometrium compared to secretory and post-menopausal endometrium

**Figure 2:**
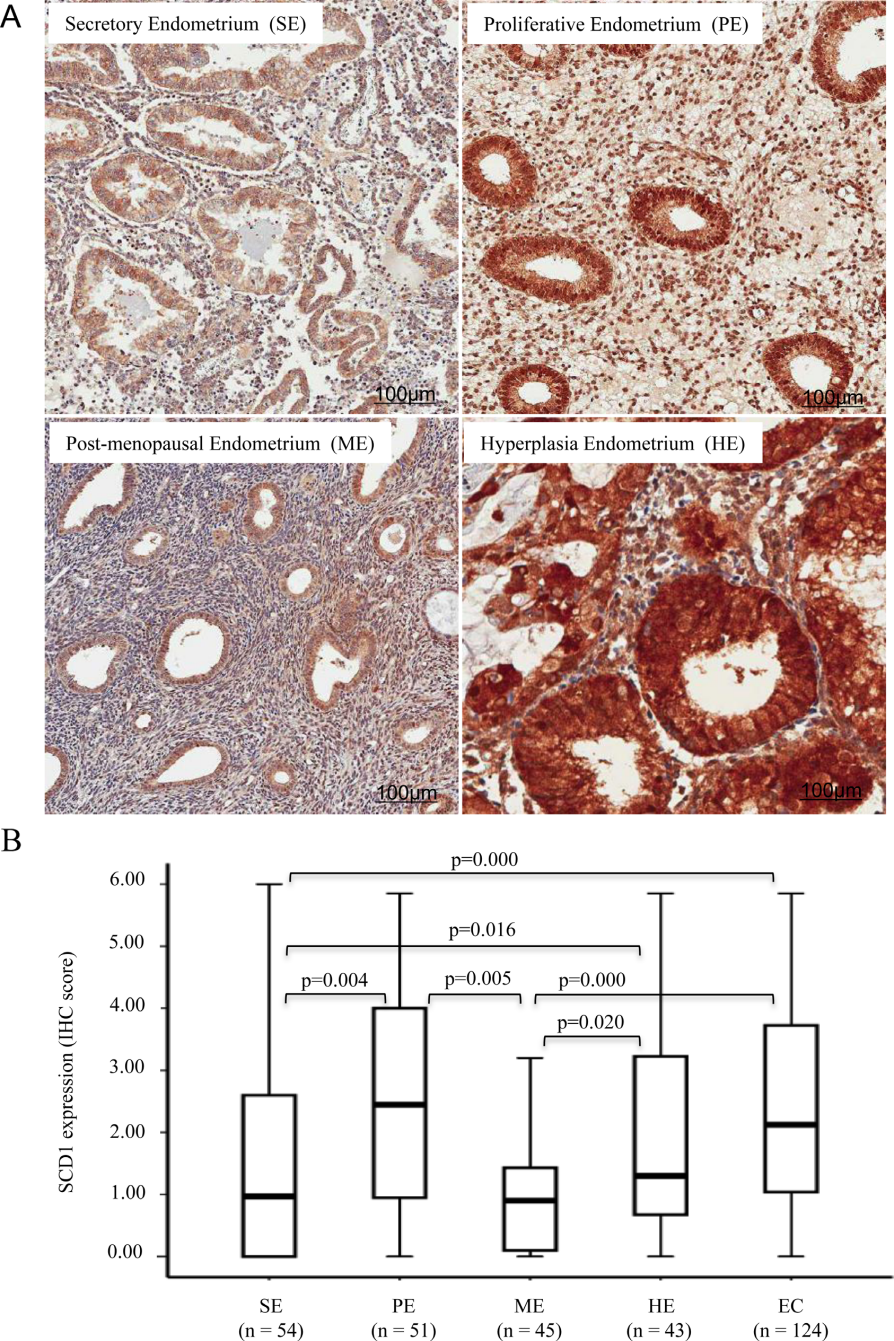
SCD1 expression is increased in proliferative and hyperplasia endometrium (**A**) IHC staining was conducted with anti-SCD1 antibody on endometrial tissues derived from normal and atypical hyperplasia endometrium. Secretory, proliferative and post-menopausal normal endometrial tissues were stained. (**B**) Boxplot of IHC staining score for SCD1 expression in whole cell in normal, hyperplastic and cancerous tissues in all specimens recruited to this study as indicated. Statistical analysis of SCD1 expression was performed, and showed the *p*-value for the significant difference among the experimental groups (*p* < 0.05).

Given that the incidence and progression of endometrial carcinoma was associated with the menopausal status, we investigated the expression level of SCD1 in normal cyclical endometrium and post-menopausal endometrium. Throughout menstrual cycle, the endometrium presents a wide spectrum appearance due to the cyclic steroid hormone levels. As shown in Figure 2A, SCD1 was highly expressed in epithelial cells of proliferative endometrium compared to the secretory endometrium. The observation of increased SCD1 expression in proliferative endometrium was consistent with the notion that the rapid proliferating cells in endometrium required *de novo* lipogenesis [29]. SCD1 was weakly expressed in post-menopausal endometrial epithelial cells, as well as in the interstitial cells regardless of the endometrial cyclical status (Figure 2). Endometrial atypical hyperplasia is a precancerous condition. We performed immunohistostaining of SCD1 in atypical hyperplasic endometrial tissues. As shown in Figure 2A and 2B, SCD1 expression was significantly increased in hyperplasic endometrium compared to normal endometrium. These findings indicate that SCD1 upregulation occurs in the endometrial precancerous lesions, which may contribute to the development and progression of endometrial carcinaoma.

### SCD1 is required for cell proliferation

**Figure 3:**
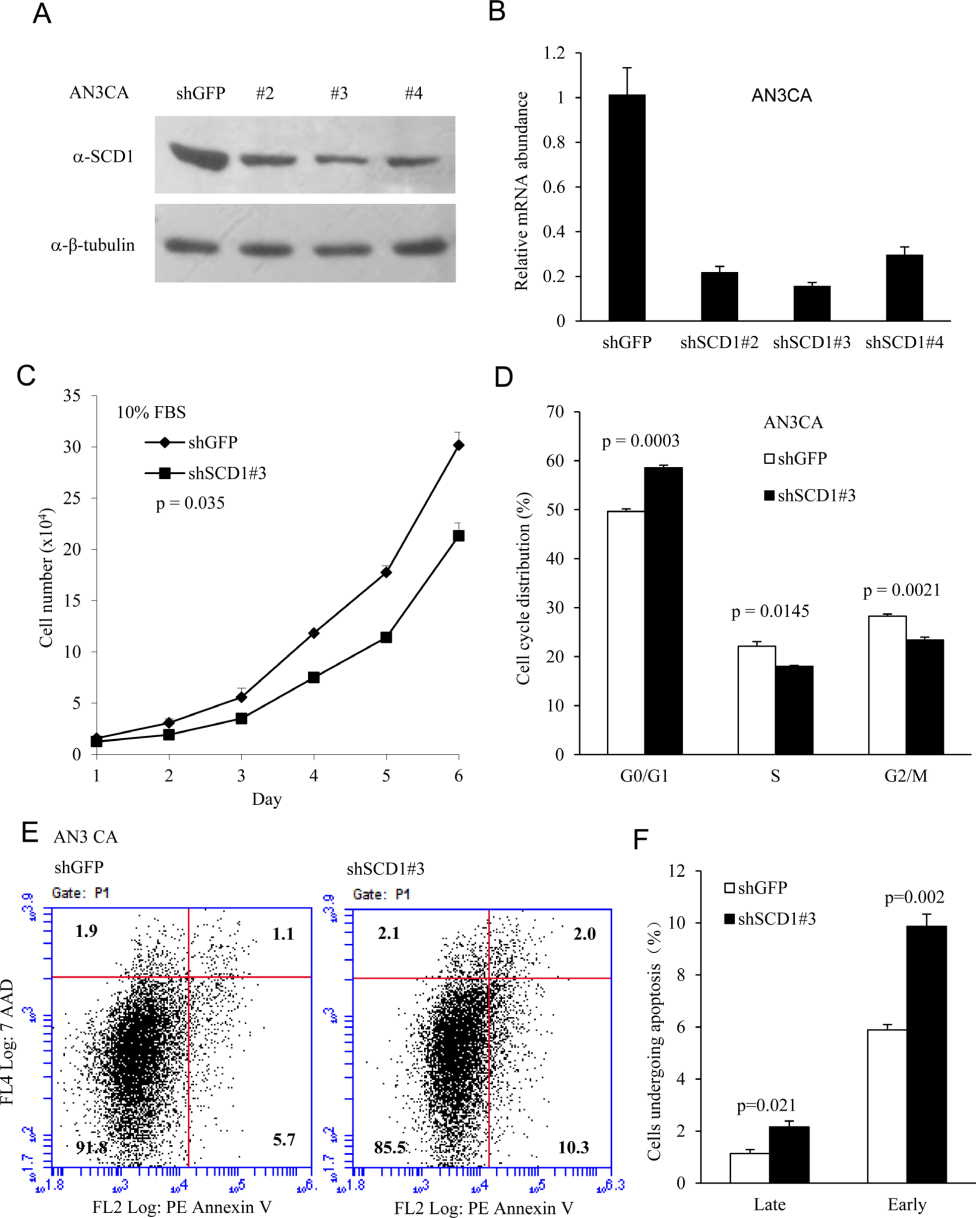
SCD1 is required for cell proliferation in endometrial cancer cells Endometrial cancer AN3 CA cells were transduced with a set of lentiviral vector expressing short hairpin RNA targeting SCD1 gene. Western blot (**A**) and qRT-PCR analysis (**B**) of SCD1 were performed in AN3 CA cells showing a successful knockdown of SCD1. (**C**) 1 × 10^4^ of AN3 CA cells were seeded per well in a 12-well cell culture device. Cellular growth was determined by counting the cells at different time points as indicated. Cells with knockdown of endogenous SCD1 were partially defective for cell growth. (**D**) The effects of SCD1 reduction on cell cycle progression of the AN3 CA cells analyzed by flow cytometry analysis. (**E**) AN3 CA cells transduced with shSCD1 and control vector were subjected to Annexin V analysis for apoptotic cell death. Knockdown of SCD1 promotes cell death. (**F**) Late and early apoptotic cell death was shown as percentage to the total cells counted.

Biosynthesis of MUFA was shown to be very important in mitogenic signaling or oncogene-induced cell proliferation. Because SCD1 regulates the lipid metabolic process that provides cancer cells with MUFA, an important precursor of phospholipids, we hypothesis that knockdown of endogenous SCD1 would restrain cell proliferation and growth. To identify the importance of SCD1 in the proliferation of cultured EC cells *in vitro*, we detected the effects of SCD1 knockdown on cell growth. As shown in Figure 3A and 3B, short hairpin RNA targeting SCD1 gene achieved successful knockdown efficiency. Importantly, SCD1 knockdown impaired cell proliferation in AN3 CA cells treated with 10% FBS (Figure 3C), while the difference was more significant in cells treated with 10% lipid-free FBS (Supplementary Figure 1A), demonstrating that SCD1 is indispensable for EC cell proliferation. Next, to determine whether SCD1 is essential for cell-cycle progression, we performed FACS analysis, and found that depletion of SCD1 reduced the cells in S and mitotic phases, and increased cell numbers in G1 phase (Figure 3D), indicating that elevated levels of SCD1 may facilitate G1/S transition.

### Downregulation of SCD1 induces apoptotic cell death

The tumor growth is resulted from the net gain of cell number, which is determined by cell proliferation and death. It was previously shown that inhibition of SREBP1 sensitized tumor cells to death ligand [30], and SREBP1 depletion led to increased cell death [28]. To detremine whether SCD1 is essential for cell survival, we downregulated SCD1 by shRNA and detected the apoptotic cell death by annexin V staining, which could be a marker of early cell death [31]. Cells were stained with Annexin V and 7-AAD. As shown in Figure 3E, 3F), the early cell death marked by Annexin V-positive and 7-AAD-negative increased from 5.7% to 10.3%, respectively, in AN3 CA cells with decreased SCD1 expression. Cells in late apoptosis (both Annexin V and 7-AAD positive) were low in all these cells, but showed an increase from 1.1% in vector control cells to 2% in SCD1 downregulated cells.

### Knockdown of SCD1 impairs lipids formation and colony-forming capacity of EC cells

**Figure 4:**
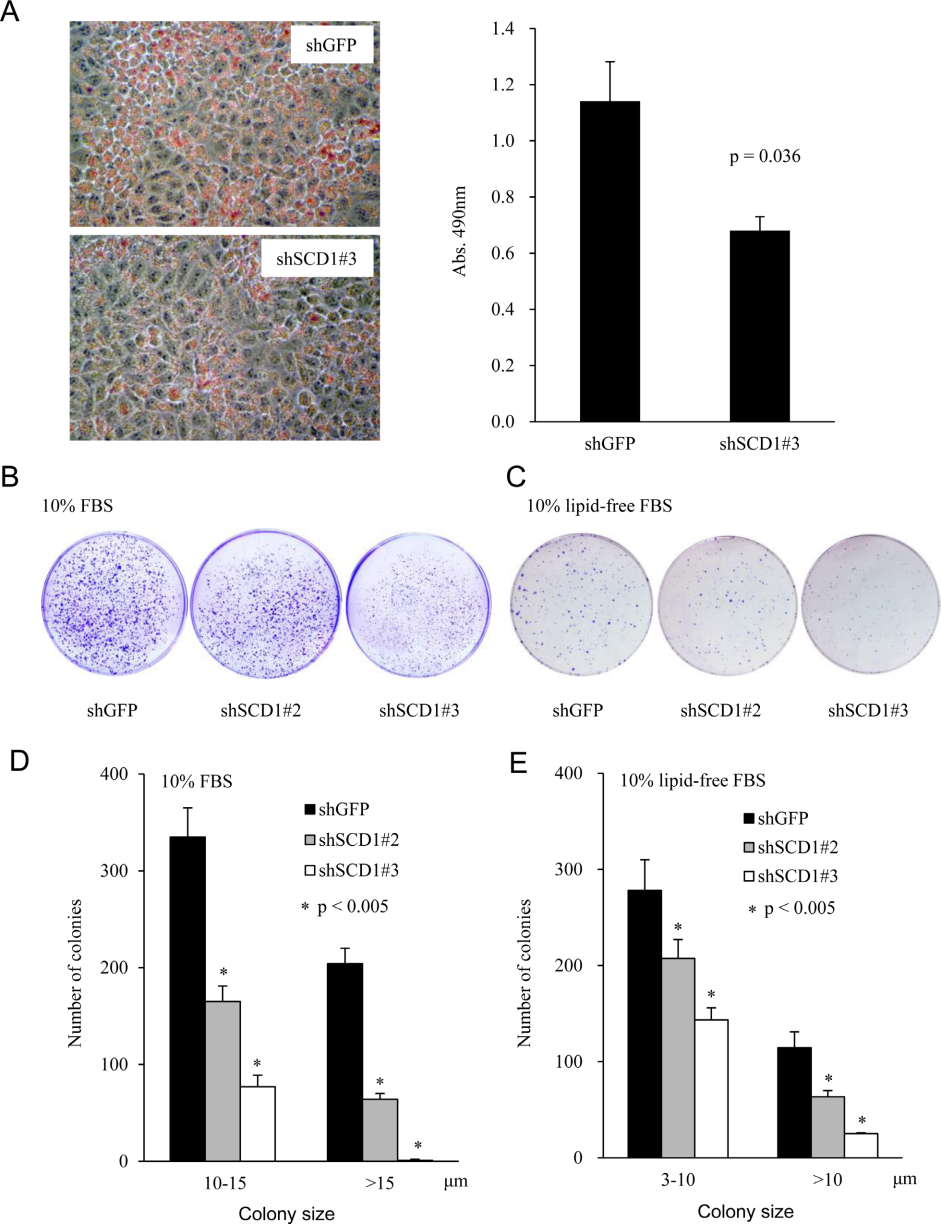
Knockdown of SCD1 expression represses lipid production and foci formation of endometrial cancer cells (**A**) Oil Red O staining of cells stably expressing shRNA targeting SCD1 or vector control (left panel). The staining was quantitatively determined (right panel). (**B** and **C**) A total of 1,000 cells were seeded in 6-cm cell culture dish, and allowed to grow for 2 weeks. Medium was replaced every three days with fresh MEM supplemented with either normal FBS (B) or lipid-depleted FBS (C). Cells were stained with crystal violet. (**D** and **E**) The number and size of colonies were counted and calculated using software Gel-pro Analyzer.

SCD1 is a rate-limiting enzyme to produce unsaturated fatty acids. To determine whether SCD1 knockdown impairs EC cells’ ability to synthesize lipids, we performed Oil Red O staining. As shown in Figure 4A, reduced staining was observed in shSCD1 cells compared to the vector control cells (shGFP), suggesting that SCD1 knockdown significantly reduced the lipid formation (Figure 4A, right panel). In addition, we implemented the clonogenic assay to determine the capacity of SCD1 in tumor colony formation. As shown in Figure 4B, SCD1 downregulation by shRNA significantly decreased the number of colonies formed in 6-cm culture dish. The protein level of SCD1 was downregulated almost 80% in the AN3 CA shRNA#3 cells, which reduced the large colonies (size > 15 μm) by 100%, while the AN3 CA shRNA#2 cells, by reducing SCD1 expression by 60% (Figure 3A), restrained the colony-forming capacity of AN3 CA cells by 30% (Figure 4D). Given cells may uptake lipids from serum in cell culture. We also performed the experiments in medium supplemented with lipid-depleted serum, which led to similar observations (Figure 4C, 4E). These results suggest that the colony formation ability of EC cells positively correlates with the levels of the SCD1 expression, and that *de novo* synthesis of lipids is indispensable for EC tumorigenesis.

### SCD1 is indispensable for tumor growth *in vivo*

**Figure 5:**
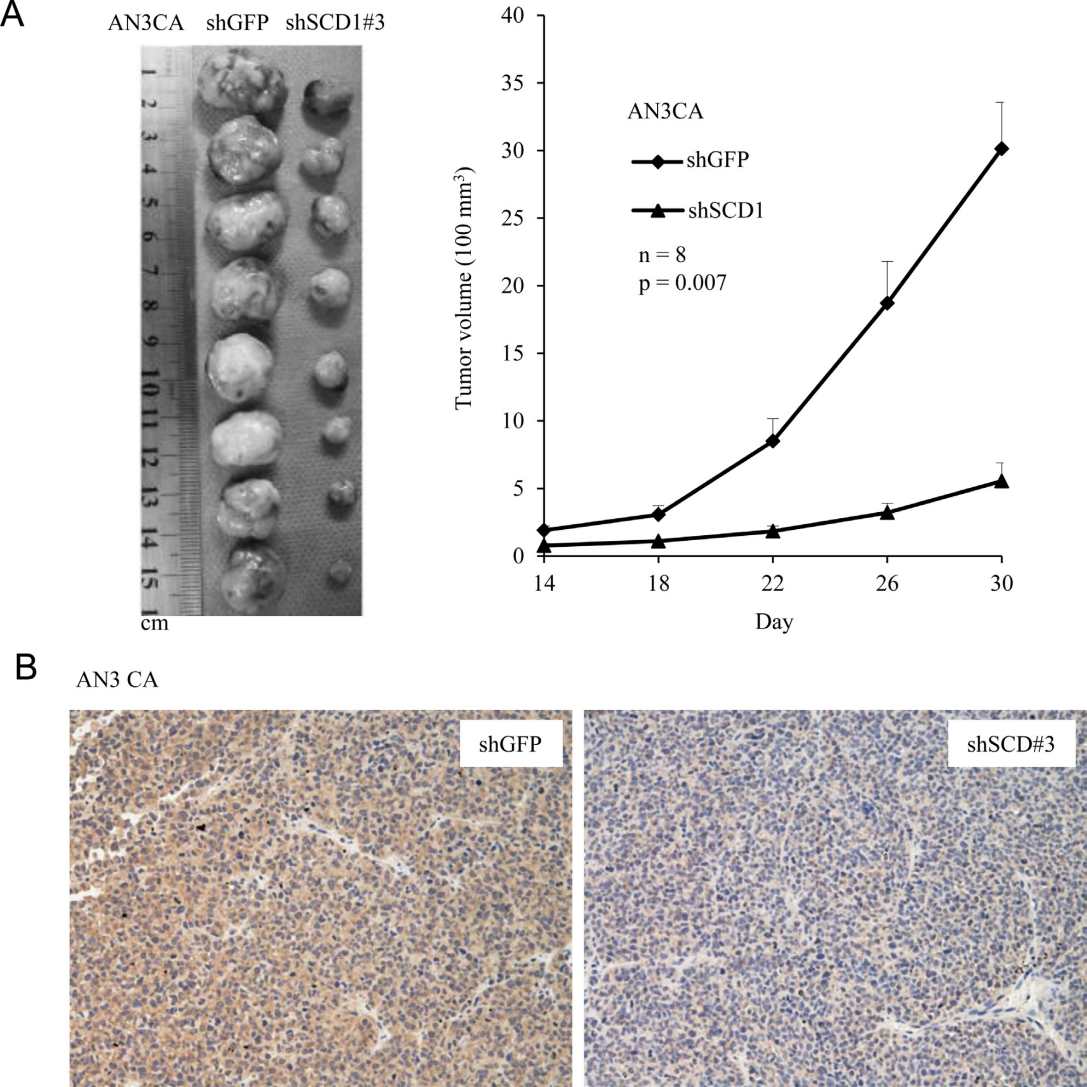
SCD1 is required for tumorigenesis in AN3 CA cells (**A**) AN3 CA cells (5 × 10^6^) either knockdown of SCD1 or vector control were implanted subcutaneously in two flanks of each SCID mice. Tumor growth was measured every four days by using a digital caliper and tumor volume was calculated. (**B**) IHC was conducted on tumor tissues collected from mice using anti-SCD1 antibody.

To examine the contribution of SCD1 to tumor growth *in vivo,* we exploited a xenograft SCID mouse model. A total of 5 × 10^6^ of SCD1 knockdown cells (shRNA#3) were subcutaneously implanted to each mouse, then we measured tumor size every four days to monitor tumor growth. As shown in Figure 5A, AN3 CA cells with depletion of SCD1 by shRNA significantly decreased the tumor sizes compared to the vector control cells. In addition, tumor tissues were examined by IHC using anti-SCD1 antibody, and the results further confirmed the knockdown efficiency *in vivo* (Figure 5B). These findings are in accordance with the observation that SCD1 knockdown cells had reduced growth rates compared to the vector control cells (Figure 3C). Collectively, these results demonstrate that SCD1 is indispensable for tumor growth *in vivo*.

### Pharmaceutical blockage of SCD1 activity inhibits EC cell proliferation and colony formation

**Figure 6:**
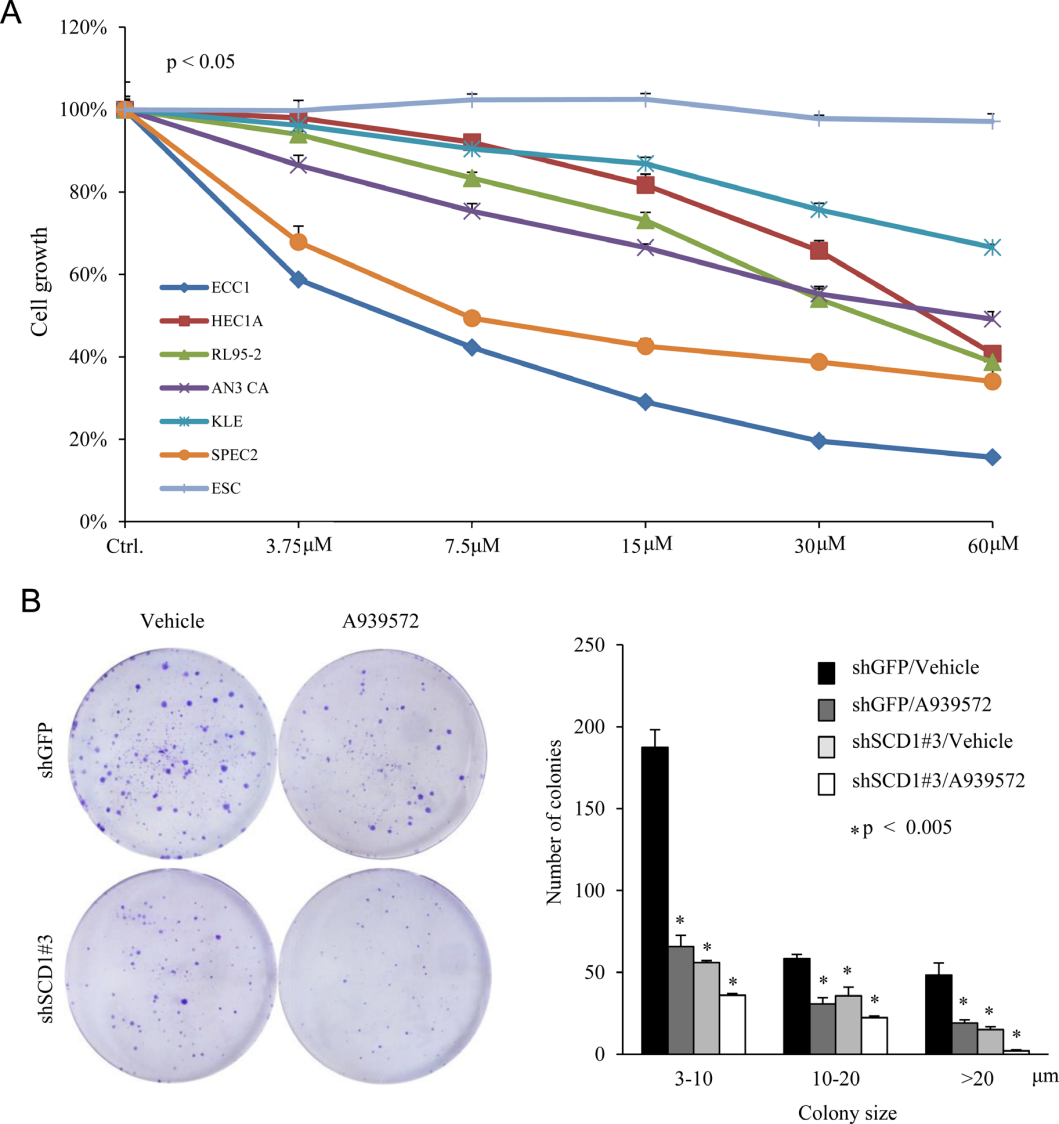
SCD1 inhibitor represses endometrial cancer cell growth and colony formation (**A**) Endometrial cancer cells were treated with increased dose of SCD1 inhibitor A939572. Cell growth was determined by cell counting. Endometrium stromal cells (ESC) serves as a control. (**B**) Foci formation experiment was conducted on AN3 CA cells stably expressing shSCD1 or vector in the presence and absence of a 10 μM dose of A939572.

Using shRNA approach, we established that SCD1 could serve as a therapeutic target to block EC growth. Next, we tested whether chemical inhibitors of SCD1 could inhibit EC cell proliferation. We examined a set of commonly used EC cell lines, and confirmed that all have detectable levels of SCD1 protein expression (Supplementary Figure 1B and 1C). A939572 is a small molecular compound which has been demonstrated to inhibit SCD1 enzymatic activity [32]. We found that A939572 inhibited the growth of all endometrial cancer cells in a dose-dependent manner, but had no significant effect on endometrial stromal cells (ESC) (Figure 6A). EC cells treated with a 10 μM dose of A939572 showed significant reduced colony formation ability, similar to those SCD1 knockdown EC cells (Figure 6B). These results demonstrate the feasibility to use pharmaceutical inhibition of SCD1 as a therapy to repress EC growth and progression.

## DISCUSSION

Cancer cells are distinct from nonmalignant cells for its unusual requirement for fatty acid synthesis [6]. In growing tumors, energy supply is principally provided by lipids coming from *de novo* synthesis rather than from circulating lipids [33]. Several observations have demonstrated the importance of fatty acid metabolism in cell transformation and tumor progression [34]. Increased lipogenesis is considered a hallmark of cancer progression and metastasis.

The increased lipogenesis in cancer is reflected by the overexpression and hyperactivity of lipogenic enzymes, such as FASN and SCD1. A large number of cancer cells contain higher levels of MUFAs suggesting a role for SCD1 in tumorigenesis. Previous studies demonstrate that SCD1 functions as an oncogenic factor in certain types of cancers [35–41]. First of all, Overexpression of SCD1 appears to be correlated with tumor aggressiveness [35], and may serve as a prognostic biomarker [36]. Secondly, SCD1 promotes colon cancer cell proliferation by facilitating β-catenin activated gene expression [37]. Furthermore, SCD1 is closely associated with tumor promotion, late stage and poor survival in lung cancer [38]. Moreover, SCD1 upregulation may shorten the survival time of breast cancer patients. Knock-down of SCD1 impairs β-catenin signaling and inhibits epithelial-mesenchymal transition-like behavior of metastatic breast cancer cells [39]. Finally, we and others [37–41] indicate that loss of SCD1 activity yields pronounced viability inhibition of various cancer cells *in vitro*.

These findings demonstrate SCD1 upregulation both transcriptionally and at the protein level (Figure 3). The mRNA expression of SCD1 is dependent on many factors, including diet, hormones, and the activity of other genes. In actual, the levels of mammalian SCD1 appear to be basically determined by its rate of transcription. In this respect, we need further efforts to clarify the potential regulatory mechanism. We previously reported that SREBP1 was indispensable for the tumorigenesis of endometrial carcinoma [28]. Our observation illuminated that SREBP1 was overexpressed in endometrial cancer patients, and its activation was correlated with high grade and poor differentiation of EC [28]. In the current study, we further show that one of SREBP1 downstream target genes, SCD1 is indispensable for EC cell survival and proliferation.

SCD1 is a major target of growth factors and hormones that regulate pivotal cell cycle events [42]. A large number of potent mitogens (such as epidermal growth factor, insulin) have been shown to stimulate SCD1 expression in several human untransformed cell types [43–45]. Furthermore, the upregulation of SCD1 in cancer cells could prevent the cytotoxic effects of SFA.

In two breast cancer cells, MCF7 and MDA-MB-231, SCD1 downregulation does not affect cell viability 48 hours post-transfection. However, in several other cancer cells, decreasing SCD1 expression affects cell viability [36–39]. In our study, SCD1 inhibitor has no significant effect on cell viability of normal endometrial stromal cells (ESC) with the increase of drug concentration (Figure 6A). SCD1 downregulation significantly impaired cell proliferation in AN3 CA cells treated with 10% FBS, while the difference was more significant in cells treated with 10% lipid-free FBS (Supplementary Figure 1A), The difference in cell sensitivity to SCD1 inhibition could be attributed to the presence of certain oncogene pathways or a higher concentration of MUFA in the media. SCD1 depletion does not universally affect cell proliferation for the ECC1 cells are more affected by SCD1 inhibition than the KLE cells. We hypothesis that it is correlated with the SCD1 expression level and other growth factors, the cancer cell types and serum components might also influence the effect of SCD1 inhibition on cell viability.

Inhibition of the protein expression or activity of lipogenic enzymes with either short hairpin RNA or small molecule inhibitors impairs cancer cell viability in *in vitro* and *in vivo* [46–50]. In particular, targeting FASN with chemical inhibitors has been proved to be effective in repressing tumor growth [51]. In addition, knockdown of SREBP1, the major regulator of lipogenesis which controls the expression of *FASN* and *SCD1*, efficiently inhibited tumor growth. Our results demonstrate that SCD1 is required for supporting EC cell growth and survival, indicating that SCD1 may represent a novel target to repress EC growth and progression. Several other specific small molecule inhibitors of SCD1 activity have been recently discovered [52], however, the utility of these SCD1 inhibitors as potential agents for chemotherapy deserves further validation. The antigrowth effect of SCD1 blockade proves to be selective for cancer cells, this may offer the possibility of interventional window for SCD1 inhibitors in cancer treatment.

In summary, we have demonstrated that SCD1 is significantly upregulated and functionally activated in clinical EC specimens, and that SCD1 plays an important role in EC growth and progression. Data from both *in vitro* and *in vivo* models consistently indicate that upregulation of the endogenous SCD1 is required for EC cell growth, colony formation and tumor growth. These results suggest that SCD1 represents a novel and valid therapeutic target in treating EC, which warrants the clinical trials with SCD1 chemical inhibitors in the future.

## MATERIALS AND METHODS

### Endometrial cancer specimens and immunohistochemistry (IHC) staining

Formalin-fixed and paraffin-embedded tumor specimens used in this study were from the tissue bank of the 90th Hospital of Jinan, China. All tumors were primary and untreated before surgery with complete clinicopathological information. All of the specimens were anonymous and tissues were collected in compliance with institutional review board regulations. Patients with endometrial cancer and tumor characteristics for this study population were reported in our prior study [28]. Endometrial cancer tissues from the 90th Hospital of Jinan were built into a 60-core array with 2 mm diameter of the core size. Adjacent normal tissues were included for some cancer tissues.

IHC staining for SCD1 expression was conducted as described previously [28]. Hematoxylin and eosin (H&E) stainings were reviewed to ensure the cancer tissue and normal epithelium. All of staining was assessed by pathologists blinded to the origination of the samples using a semi-quantitative method. Tissue was scored (H-score) based on the total percentage of positive cells and the intensity of the staining (1+, 2+ or 3+), where H = (% “1+” × 1) + (% “2+” × 2) + (% “3+” × 3). A minimum of 100 cells was evaluated in calculating the H-score.

The statistical analyses were performed using SPSS software (Version 13.0, Chicago, IL, USA). Means of continuous variables for SCD1 staining intensity between endometrial cancer and each normal endometrial phase or within normal endometrial phases were compared by one-way analysis of variance (multiple comparisons). The comparison between the clinicopathologic characteristics of endometrial cancer and SCD1 staining intensity was evaluated with the Mann-Whitney *U* test. All statistical tests were two-sided, and *p*-values less than 0.05 were considered as statistically significant.

### Plasmids, antibodies and reagents

A set of seven shRNAs targeting human SCD1 were purchased from Open Biosystems. Rabbit anti-SCD1 (MC38, C12H5) antibodies were purchased from Cell Signaling Technology (Beverley, MA, USA), and rabbit anti-β-tubulin antibody was from ZSGB-BIO (Beijing, China). Methyl thiazolyl tetrazolium (MTT), dimethyl sulfoxide (DMSO), propidium iodide, and protease inhibitor cocktail were purchased from Sigma-Aldrich (Sydney, NSW, Australia). SCD1 inhibitor (A939572) was purchased from MedChem Express (Shanghai, China). Trizol, the First Strand cDNA synthesis kit and primers were purchased from Invitrogen (Waverley, VIC, Australia). Fetal bovine serum (FBS) was obtained from GIBCO (Brooklyn, VIC, Australia).

### Cell culture

Endometrial cancer cell lines, including ECC-1, HEC-1A, RL95-2, KLE and AN3 CA, and human embryonic kidney 293T cells (HEK 293T) were purchased from American Type Culture Collection (ATCC). The basal culture medium are RPMI-1640 (ECC-1), McCoy’s 5a (HEC-1A), DMEM/F12 (RL95-2, KLE), Eagle’s Minimum Essential Medium (AN3 CA) and DMEM (HEK 293T) according to ATCC. Ishikawa cells, established in 1985 by Nishida *et al.* [53] from an endometrial adenocarcinoma that was ER-positive and PR-positive, were maintained in DMEM-F12 containing 1% penicillin/streptomycin and supplemented with 10% fetal bovine serum (FBS) at 37°C in a humidified atmosphere of 5% CO_2_. The progestin-resistant Ishikawa (PR Ishikawa) cells were induced by medroxyprogesterone acetate in 2006 by Zhao *et al.* [54] for more than 10 months. Cultures were passaged every 2–3 days depending on the cell lines, using 0.25% Trypsin-EDTA (Invitrogen) for cell detachment. Type II endometrial cancer cell lines, SPEC-2 was a gift from Wenxin Zheng at the University of Arizona College of Medicine [55]. Endometrium stromal cells (ESC) were separated by digesting the minced tissues as previously described [56]. Under lipid-free culture condition as indicated, the basal medium was supplemented with 10% lipid-depleted FBS purchased from Cocalico Biologicals (#55-0116).

### Cell transfection and transduction

For transient transfection, Lipofectamine 2000 (Invitrogen) was used according to manufacturer’s protocol. For cell transduction, lenti-viruses were prepared using Trans-Lentiviral shRNA Packaging Kit following manufacturer’s instruction (Open Biosystems) with modifications. Briefly, lenti-viral vector expressing shRNA will be introduced into HEK 293T cells by transient co-transfection with helper virus with calcium phosphate precipitation. After 6 hours, cell culture medium was replaced, and cells were allowed to grow for 36 hours to produce viruses. The supernatant was then collected and filtered through a 0.45-μm filter. Cells were infected at approximately 70% confluence in culture medium supplemented with 8 μg/ml polybrene. After 48 hours later, AN3 CA cells were stably selected by supplementing the medium with 2 μg/ml puromycin for 2 weeks. The efficiency for knockdown was determined by western blot and qRT-PCR assays.

### Western blot

The parental Ishikawa, progestin-resistant Ishikawa, AN3-CA, ECC-1, HEC-1-A, RL95-2, KLE and SPEC-2 cells were plated in 6-cm dishes. At 80%–90% confluence, cells were rinsed 2 times with cold phosphate-buffered saline (PBS) and then harvested using a cell scraper. Total protein was extracted from cultured cells or frozen tissues using RIPA lysis buffer. Western blot was performed as we previously described with antibodies indicated. Chemo-luminescence was detected with BeyoECL Plus reagent (Beyotime, Shanghai, China) and visualized using the Fujifilm LAS-3000 imaging system (Bundoora, VIC, Australia).

### Quantitative real-time PCR (qRT-PCR)

Total RNA was isolated from cultured cells using Trizol Reagent (Invitrogen) following manufacturer’s instructions. RNA (2.0 μg) was subjected to reverse transcription to synthesize cDNA using the SuperScript^™^ II Reverse Transcriptase Kit (Invitrogen). Quantitative PCRs were then carried out with SYBR Green PCR Master Mix (TIANGEN Biotech Co., Beijing, China) in a real-time PCR System (Applied Biosystems 7500, Carlsbad, CA, USA) following standard procedure. For qRT-PCR, each reaction (20 μl) consisted 1 μl reverse transcription cDNA product and 100 nM of each primer. The primers used for qRT-PCR were obtained from the PrimerBank database (http://pga.mgh.harvard.edu/primerbank/). The PCRs proceeded under the following conditions: 95°C for 15 minutes, followed by 40 cycles at 95°C for 10 seconds, 55°C for 30 seconds and 72°C for 32 seconds. The endogenous controls used for normalization were glyceraldehyde-3-phoshate dehydrogenase (GAPDH). Gel electrophoretic analysis of qRT–PCR products confirmed that the primers amplified a single band with the expected size.

SCD1 primer (amplicon size: 116 bp): Forward sequence: TTCCTACCTGCAAGTTCTACACC; Reverse sequence: CCGAGCTTTGTAAGAGCGGT

GAPDH primer (amplicon size: 101 bp): Forward sequence: ACAACTTTGGTATCGTGGAAG; Reverse sequence: GCCATCACGCCACAGTTTC

### Oil red O staining

Cells were plated at 70% confluence and allowed to grow until 100% confluence for two days. The staining and quantification were performed as previously described [57]. Data were presented as mean ± SEM from triplicates.

### Cell viability assay and proliferation assay

Cells (1 × 10^4^) were plated in 96-well plates for 24 hours and then treated with dimethyl sulfoxide or varying doses of A939572 for 72 hours. Cell viability was determined by adding 10 μl of 5 mg/ml MTT. After 4 hours incubation in incubator, old medium was discarded, and added 100 μl of DMSO. The absorbance was determined at 490 nm using a microplate reader, and the percentage absorbance was calculated against dimethyl-sulfoxide-treated cells. For cell proliferation assays, cells were stably transfected with shRNA targeting SCD1 and control were seeded at a density of 1.0 × 10^4^ cells per well in 12-well culture device in MEM containing 10% FBS or lipid-free FBS respectively. The total number of cells per well was counted for 6 days. Each condition was replicated in triplicate (mean ± SEM).

### Foci formation assay

For colony formation assays, a total of 1.0 × 10^3^ cells were seeded in 60-mm plates or 6-well plate and allowed to grow for two weeks. The culture medium was replaced every three days with fresh MEM supplemented with either regular FBS with or without DMSO or SCD1 inhibitor (10 μM A939572) or lipid-depleted FBS. Each condition was replicated in triplicate. The number of colonies formed per plate was stained with crystal violet and quantified by using a Gel-Pro Analyzer (Media Cybernetics, Inc.). Each condition was replicated in triplicate.

### Cell cycle and cell death assays

Cells were plated at a density of 1.5 × 10^6^ per 60-mm dish and allowed to grow for 24 h, after which the medium was changed to serum-free medium. After 24 horrs of serum starvation, cells were released to regular completed medium for another 24 hours. Then cells were harvested by trypsinization, washed twice with PBS, and pelleted by centrifugation for 5 min at 500 × g. Cells were then resuspended in PBS, fixed with 75% cold ethanol overnight at 4°C, washed twice with PBS, and subsequently resuspended in 1 ml of 50 μg propidium iodide solution with 50μg RNase A and incubated for 30 minutes avoid of light before analysis. Cells were next filtered through a 100-mesh filter, and a total of 20,000 stained nuclei were analyzed with ACCURI C6 flowcytometer (BD Biosciences, San Jose, CA, USA) and CFlow Plus analysis software. Cell death was determined by using PE AnnexinV/7 AAD Apoptosis Detection Kit (BD Biosciences) following manufacturer’s instructions. Data were presented as mean ± SEM from triplicates.

### *In vivo* xenograft model

All animal experiments were performed following animal protocols, which have been approved by the Institutional Animal Care and Use Committee (IACUC) at Peking Union Medical College. AN3 CA cells (5 × 10^6^) with either knockdown of SCD1 or vector control were implanted by subcutaneous injection in two flanks of 5–6 -week-old female SCID mice. Comparisons were made for 8 animals in each group between AN3 CA/vector and AN3 CA/shSCD1#3. The tumor growth rates were examined using serial caliper measurements. The tumor volume were calculated using the equation (a × b^2^)/2 where “a” and “b” are length and width of the tumor, respectively. At the completion of the experiments, tumors were excised and statistical significance of differences in tumor volume was logarithm transformed and analyzed using a linear mixed model. Data prior to day 14 were ignored due to zeroes at day 0 (inability to take logarithms) and an initial nonlinearity or change in some of the animals growth patterns prior to day 14. Thus, the intercept at day 12 is interpretable as initiation of growth, and the slope is interpretable as rate of growth.

## SUPPLEMENTARY MATERIALS FIGURE



## Abbreviations

EC: endometrial cancer
SCD: Stearoyl CoA desaturase
MUFA: monounsaturated fatty acids
SFA: saturated fatty acids
SREBP1: sterol regulatory element-binding protein 1
PPAR: Peroxisome Proliferator Activated Receptor
FASN: fatty acid synthase
shRNA: short hairpin RNA
FBS: fetal bovine serum
