# Supplementary material for: Targeting stearoyl-CoA desaturase 1 to repress endometrial cancer progression
Source: Oncotarget. 2018 Jan 24;9(15):12064–78. doi: 10.18632/oncotarget.24304 (PMC5844729; doi:10.18632/oncotarget.24304)
Supplement: Supplementary file 1 [file oncotarget-09-12064-s001.pdf]

Targeting stearoyl-CoA desaturase 1 to repress endometrial cancer progression

SUPPLEMENTARY MATERIALS

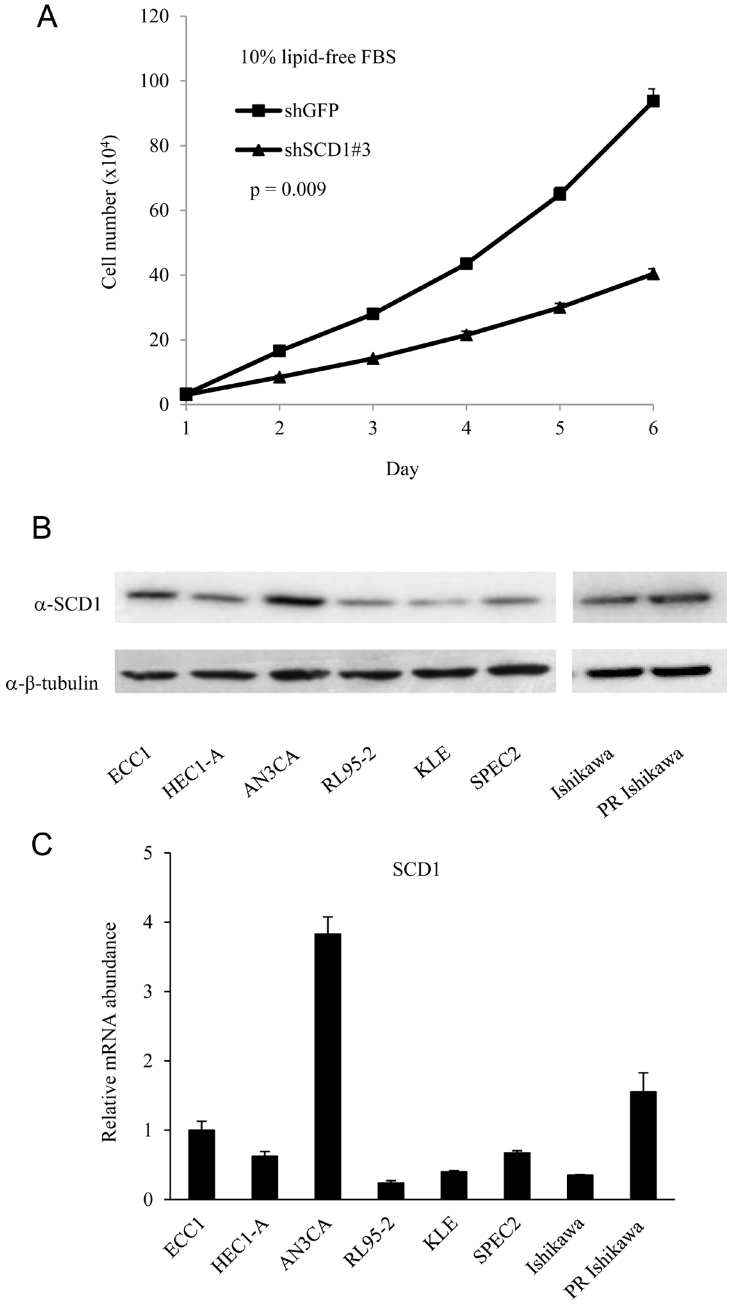

Supplementary Figure 1: (**A**) Cells with knockdown of endogenous SCD1 were partially defective for cell growth. The difference was more significant in cells treated with 10% lipid-free FBS. (**B**) Western blotting (WB) analysis of SCD1 expression in commonly used endometrial cancer cell lines. β-tubulin serves as protein loading control. (**C**) Quantitative RT-PCR analysis of mRNA abundance of SCD1 in endometrial cancer cells. RNA abundance was shown as fold change relative to that in ECC1 cells.
